# Occupational Lifting, Fetal Death and Preterm Birth: Findings from the Danish National Birth Cohort Using a Job Exposure Matrix

**DOI:** 10.1371/journal.pone.0090550

**Published:** 2014-03-10

**Authors:** Emina Mocevic, Susanne Wulff Svendsen, Kristian Tore Jørgensen, Poul Frost, Jens Peter Bonde

**Affiliations:** 1 Department of Occupational and Environmental Medicine, Bispebjerg Hospital, University of Copenhagen, Copehagen, Denmark; 2 Danish Ramazzini Centre, University Department of Occupational Medicine, Herning Regional Hospital, Herning, Denmark; 3 Danish Ramazzini Centre, Department of Occupational Medicine, Aarhus University Hospital, Aarhus, Denmark; Baylor College of Medicine, United States of America

## Abstract

**Objective:**

We examined the association between occupational lifting during pregnancy and risk of fetal death and preterm birth using a job exposure matrix (JEM).

**Methods:**

For 68,086 occupationally active women in the Danish National Birth Cohort, interview information on occupational lifting was collected around gestational week 16. We established a JEM based on information from women, who were still pregnant when interviewed. The JEM provided mean total loads lifted per day within homogeneous exposure groups as informed by job and industry codes. All women were assigned an exposure estimate from the JEM. We used Cox regression models with gestational age as underlying time variable and adjustment for covariates.

**Results:**

We observed 2,717 fetal deaths and 3,128 preterm births within the study cohort. No exposure-response relation was observed for fetal death, but for women with a prior fetal death, we found a hazard ratio (HR) of 2.87 (95% CI 1.37, 6.01) for stillbirth (fetal death ≥22 completed gestational weeks) among those who lifted >200 kg/day. For preterm birth, we found an exposure-response relation for primigravid women, reaching a HR of 1.43 (95% CI 1.13, 1.80) for total loads >200 kg per day. These findings correspond to an excess fraction of 11% for stillbirth and 10% for preterm birth.

**Conclusion:**

We found an increased risk of stillbirth among women with a prior fetal death, who lifted >200 kg/day, and an exposure-response relationship between occupational lifting and preterm birth among primigravid women. The study adds to a large body of prospective studies on occupational lifting and adverse pregnancy outcomes by refined exposure assessment.

## What Is New in the Paper

- A large body of prospective studies provides reassuring evidence that occupational lifting in general is not related major risk of fetal death and preterm birth
- This study refines assessment of occupational lifting by use of information from job and industry codes
- In relation to occupational lifting, the risk of fetal death was increased in women with a prior fetal death and the risk preterm birth was moderately elevated among primigravid women. In spite of reassuring evidence that occupational lifting in general infers a small risk, there seems to be good reason to limit high levels of occupational lifting during pregnancy

## Introduction

In clinically recognized pregnancies, fetal death and preterm birth occur with a prevalence in the range of 10–14% and 5–10%, respectively [1]. Preterm birth is associated with an increased risk of perinatal and infant mortality [2] and the proportion of preterm birth seems to have increased by more than 20% among Danish women from 1995 to 2004, mostly attributable to primiparity and multiple births [3].

Among occupationally active pregnant women, the prevalence of physically demanding work is still rather high [4]. For example, 6% of pregnant women reported lifting or carrying burdens weighing >25 kg in a Dutch study [5] and 12% reported daily lifting of burdens weighing >20 kg in a Danish study [6].

The risk of miscarriage (i.e., fetal death before survival outside the uterus is considered possible) and preterm birth in relation to occupational lifting have recently been addressed in two reviews (7;8). For miscarriage, 10 epidemiological studies provided risk estimates for lifting in total ≥100 kg per day in comparison with women lifting less. The meta risk estimate was 1.32 (95% CI 0.93–1.87), but in a subset of five, the meta risk estimate was 1.02 (95% CI 0.73–1.44) [7]. For preterm birth, the median relative risk was 1.12 (interquartile range 0.90–1.30) across 17 epidemiological studies including 8 prospective cohort studies. In 11 studies with less likely bias and confounding, there was no increased risk (median relative risk 1.02, interquartile range 0.90–1.30) [8]. A formal meta-analysis of occupational lifting was not applicable because of large heterogeneity of exposure definitions across studies [8]. The review also focused on risk estimates for events appearing at different gestational ages and found no clear evidence for heterogeneity in risk related to the timing of exposures. Although this body of evidence is reassuring, the question is if true associations may have been overlooked. First, few studies have evaluated effects of lifting ≥200 kg per day, and as acknowledged by the reviewers, it therefore remains an unresolved issue if higher levels of occupational lifting confer a risk. Second, the majority of studies used individual- (as opposed to group-) based exposure assessment, which may have implied attenuation of exposure-response relations [9]. Third, exposure assessment was based upon retrospective self-reports in the majority of studies. This also partly applied to recent longitudinal studies within the Danish National Birth Cohort (DNBC), which were published after the reviews, and which reported an increased risk of fetal death [6] and preterm birth [10] with increasing total load lifted per day. Inclusion of retrospectively collected data from women who had experienced a fetal death implied a risk of overestimation of risks due to recall bias where women who already experienced pregnancy complications overestimated their lifting exposures. However, it remains to be shown if the observed risk elevations would disappear, were recall bias eliminated. Fourth, both reviews gave a lower quality assessment to studies that did not adjust for socioeconomic status, even though this might mean overadjustment due to correlations between social class and occupational lifting. Fifth, most previous studies have not evaluated spontaneous and induced preterm births separately (exceptions are Saurel-Cubizolles et al 1991 [11], Ahlborg et al 1990 [12], and Lawson et al 2009 [13]), although induced preterm birth due to severe illness of mother or foetus comprises 30–40% of preterm births [14]; inclusion of induced preterm births may have masked effects of occupational lifting on spontaneous preterm births because induced preterm births may have other causal networks than spontaneous preterm births. Sixth, the majority of previous studies have not distinguished between primi- and multigravid women. Women who have previously experienced difficulties during pregnancy or adverse pregnancy outcomes may reduce their occupational exposures when they get pregnant again, or otherwise differ from primigravid women; they may even choose not to risk another pregnancy. Thus, there is a need for exposure-response modelling using independent, group-based exposure assessment also of high levels of occupational lifting, while distinguishing between primi- and multigravid women and taking into account induced preterm births. In this study, we applied an industry and occupation specific job exposure matrix (JEM) based on means of self-reported lifting exposures within job groups. We compared self-reported exposure estimates with exposure estimates obtained using the JEM to evaluate the influence of non-occupational factors on exposure reporting in order to better understand potential bias in studies using individual-based self-reported exposure assessment. The aim of the study was to corroborate or refute the hypothesis that exposure-response relationships exist between occupational lifting and fetal death and preterm birth.

## Methods

### Ethics Statement

The DNBC has been approved by the Danish Data Protection Agency and by the National Committee on Health Research Ethics, which has also approved the consent procedure. The DNBC is a nationwide cohort of pregnant women and their offspring [15]. At their first antenatal care visit to the general practitioner in the period 1996 to 2002, women who intended to complete their pregnancy and who spoke Danish were invited to participate. The women were included in the cohort when they had signed and returned an informed consent form. The participants completed a telephone interview at on average gestational week 16. Women, who experienced a fetal death after enrollment but before the scheduled interview, were asked to answer a modified interview.

### Study population

**Figure 1:**
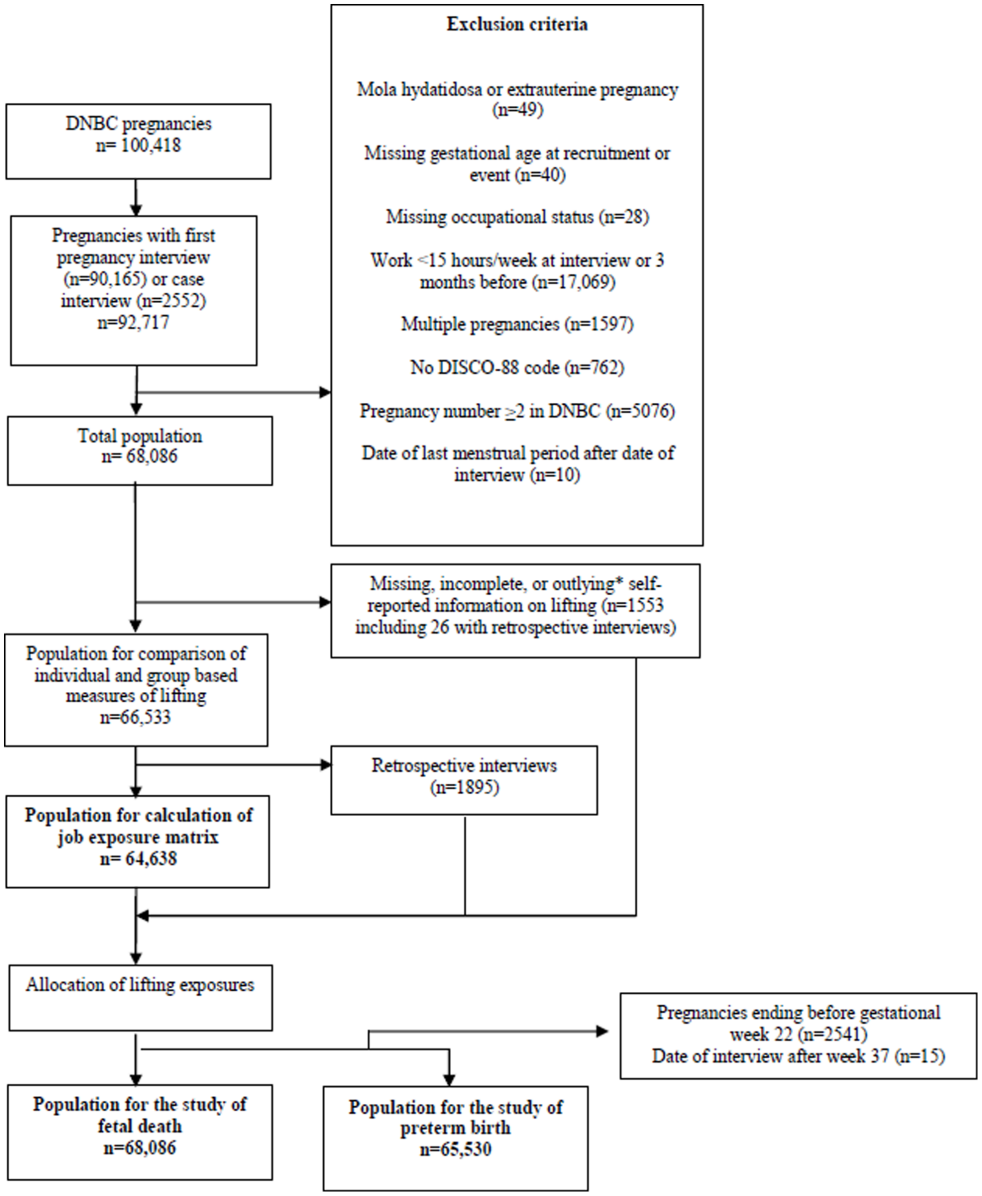
Flow chart of the study populations. * Total daily loads >10,000 kg were considered outlying (n = 12).

For each woman, we included the first pregnancy registered in the DNBC where the woman worked a minimum of 15 hours a week when interviewed or within the last three months. We excluded women with mola hydatidosa, extrauterine pregnancy, multiple pregnancies, and pregnancies with an invalid date of last menstrual period or with missing information on gestational age at recruitment or event. Furthermore, we excluded women with unknown occupational status and women without an occupational code according to the Danish version of the International Standard Classification of Occupations from 1988 (DISCO-88), Figure 1.

### Exposure assessment

The first step for construction of the JEM was grouping of jobs according to DISCO-88 and the Danish Industrial Classification of All Economic Activities obtained from Statistics Denmark. If ≥100 women had identical four digits DISCO-88 codes, they were subdivided according to industry codes. If the subdivision resulted in a group size of ≥10 women, the mean total load lifted per day was calculated and applied to all women within the group. The remaining women were allocated the mean load for groups defined by four digits DISCO-88 codes - and, again, if the group size was ≥10, the mean total load lifted per day was applied to all women within the group. Three or two digits DISCO-88 codes were used if <10 women had identical four digits DISCO-88 codes. The JEM was based entirely on prospectively collected data from the pregnancy interview, i.e., the women were pregnant when interviewed. They were asked “In your job, do you lift 11–20 kg at a time on a daily basis, i.e., less than a crate of beer and more than a bucket of water?” If they answered yes, they were asked “How many times a day do you lift 11–20 kg?” The women were also asked if they lifted more than 20 kg on a daily basis and how many times a day. For each woman, we calculated the total load lifted per day, with loads in the category 11–20 kg set to 15 kg, and loads above 20 kg set to 22.5 kg.

### Outcome measurement

Pregnancy outcomes were identified by linkage between the Danish Civil Registration System, the Danish Medical Birth Registry, and the Danish National Patient Register (DNPR). From the Danish Medical Birth Registry we obtained data on live births and stillbirths and from the DNPR we obtained information on fetal deaths and induced or spontaneous preterm births, together with information on gestational age at pregnancy termination. We classified fetal death as early fetal death (≤12 completed gestational weeks), late fetal death (13–21 completed gestational weeks), and stillbirth (≥22 completed gestational weeks) [6].

Preterm birth was defined as delivery of a live born infant after 22 and before 37 completed gestational weeks, and we classified this outcome as extremely preterm (22–27 completed gestational weeks), very preterm (28–32 completed gestational weeks), and moderately preterm birth (33–36 completed gestational weeks) [3], [10]. An induced delivery was defined by induction of labour or cesarean section before spontaneous onset of labour.

### Covariates

*A priori*, the following covariates were selected for inclusion in adjusted analyses: maternal age (15–24, 25–29, 30–34, 35–46 years), parity (nulliparous; yes/no), smoking in pregnancy (no smoking, ≤10 g of tobacco/day, >10 g tobacco/day), alcohol consumption in pregnancy (none, 0–1½ units/week, 2–3½ units/week, ≥4 units/week), and pre-pregnancy body mass index (<18.5, 18.5–24, 25–29, ≥30 kg/m^2^). For preterm birth, the following additional factors were selected: conic section before pregnancy (yes/no) and assisted reproduction (yes/no). In supplementary analyses of fetal death among multigravid women, we also included previous fetal death. We assessed socioeconomic status (SES) based on job titles and classified this variable as higher grade professional, lower grade professional, skilled worker, or unskilled worker. We only included SES in supplementary analyses because of expected high correlation with occupational lifting and because we already included other covariates related to SES [16]. Women with missing values for one or more covariates were included in the analyses in separate categories.

### Data analysis

We compared self-reported exposure estimates with exposure estimates obtained using the JEM by means of multivariable regression with bootstrap to obtain regression coefficients and 95% confidence intervals (CI). We calculated hazard ratios (HR) for the association between occupational lifting and fetal death and preterm birth using Cox regression analysis with gestational age (number of days since beginning of the last menstrual period) as underlying time variable. In the analyses of fetal death, early and late fetal deaths and stillbirths were defined as events, while live births, induced abortions, maternal death during pregnancy, and emigration were censored. In the overall analyses, follow-up started at time of consent and ended at event or censoring. The analysis of early fetal death included women who were enrolled before day 84, while still pregnant, and had an additional censoring criterion, i.e. completion of gestational week 12 (at day 84). The analysis of late fetal death included women who were enrolled before day 154, while still pregnant, with follow-up starting at day 84 or at time of enrollment, whichever came last, with completion of gestational week 21 (at day 154) as an additional censoring criterion. The stillbirth analysis included women who were enrolled after day 154, while still pregnant, with follow-up starting at day 154 or at time of enrollment, whichever came last. For fetal death, we conducted subanalyses restricted to women who had been pregnant previously, stratified by previous fetal death (yes/no). Interaction between occupational lifting and prior fetal death was tested by general linear models (SAS PROC GLM). We also conducted separate analyses for primigravid women using Cox regression analysis.

By a similar approach, the preterm birth analyses were conducted for all preterm births, and for extremely, very, and moderately preterm births. Since earlier pregnancy experience might influence the choice of a new pregnancy and exposure circumstances in a new pregnancy, we also conducted separate analyses for primi- and multigravid women. To evaluate any influence of induced preterm birth, we finally carried out analyses with censoring in case of induced birth. Both for fetal death and for preterm birth, the proportional hazards assumption was fulfilled for most of the included covariates.

We estimated the potential for prevention of fetal death and preterm birth by multiplying the excess fraction, (HR_adjusted_-1)/HR_adjusted_, for each exposure category by the number of events within the exposure category, then summing up the excess numbers across all exposure categories, and finally dividing the sum by the total number of events and converting to percent. For fetal death the estimate was based on only one exposure group (201–975 kg).

Analyses for preterm birth were performed with STATA 12 software (StataCorp LP, College Station, TX, USA) and for fetal death with SAS Statistical Software v.9.2 (SAS Institute Inc, Cary, NC, USA) on Statistics Denmark's research platform.

## Results

Characteristics of the total study population (N = 68,086) according to occupational lifting are listed in Table 1. The percentage who lifted >15 kg per day was 48%, when assessed using the JEM. Women, who lifted higher loads, were predominately unskilled workers, more likely to smoke in pregnancy, and had a higher body mass index. The subpopulation for the study of preterm birth (N = 65,530) did not differ from the total study population (results not shown). The most frequent job codes in the highest exposure category included waiters, manufacturing labourers, and transport and storage labourers accounting for 45% of the job codes in this category. Table 2 shows gestational age at recruitment and pregnancy outcomes for the total study population. For women, who experienced an early fetal death, only 4.3% of the data was collected prospectively, while for women, who experienced a late fetal death or stillbirth, these proportions were 39% and 99%, respectively.

**Table 1:** Maternal characteristics according to occupational lifting during pregnancy assessed using a job exposure matrix, N = 68,068.

	Total load lifted per day (kg)					
	Total	0–14	15–50	51–100	101–200	201–975
**N**	100	46.5	11.8	23.6	12.1	6.0
**Age at conception**						
**15–24 years**	14.9	6.0	9.5	12.4	24.7	22.0
**25–29 years**	41.3	39.8	41.5	44.4	39.9	40.7
**30–34 years**	32.7	40.5	36.3	31.9	26.3	28.7
**35–46 years**	11.1	13.7	12.7	11.4	9.2	8.6
**Pre-pregnancy body mass index**						
<18.5 kg/m^2^	4.2	4.2	4.1	3.7	4.6	4.5
18.5-<25 kg/m^2^	67.5	71.1	69.2	66.8	59.5	54.5
25-<30 kg/m^2^	19.1	17.4	17.7	19.6	22.3	26.2
≥30 kg/m^2^	7.7	5.8	7.1	8.4	11.7	13.1
**Nullipara**	49.7	49.7	46.2	48.9	49.4	59.8
**Smoking in pregnancy**						
No smoking	74.6	80.3	75.0	72.6	61.8	64.5
1–10 cigarettes/day	19.3	15.7	19.3	21.2	27.4	24.2
>10 cigarettes/day	5.8	3.8	5.5	6.0	10.6	11.1
**Alchohol consumption in pregnancy**						
None	53.7	50.0	51.8	56.3	61.0	61.7
0–1.5 units/week	33.8	35.8	34.2	33.3	29.4	29.1
2–3.5 units/week	10.0	11.6	11.5	8.4	7.6	7.0
>4 units/week	2.3	2.6	2.4	2.0	1.9	2.1
**Physical exercise in pregnancy (yes)**	37.7	39.9	38.9	37.7	33.0	27.1
**Working posture**						
Sitting	24.8	43.5	21.3	5.7	3.3	4.8
Varying	46.7	46.8	51.9	49.9	36.8	42.3
Standing/walking	28.0	9.3	26.2	43.8	59.2	51.2
**Occupational status**						
Higher grade professionals	10.8	19.6	6.7	2.2	3.1	1.0
Lower grade professionals	32.5	30.5	39.6	53.9	7.2	1.3
Skilled workers	21.7	35.4	25.9	2.5	8.9	8.4
Unskilled workers	28.7	8.3	22.6	34.9	72.1	86.4
Students	6.2	6.1	5.2	6.5	8.6	2.9
**Leisure time daily lifting >20 kg in pregnancy (yes)**	6.6	5.9	6.2	7.3	7.7	7.9
**Exposure assignment group**						
Occupational code* (2–3 digit)	0.6	0.4	0.5	0.7	1.4	1.2
Occupational code* (4 digit)	35.1	36.1	51.7	28.6	24.6	41.0
Combination of occupation and industry	64.3	63.5	47.8	70.7	74.0	57.8

Numbers in cells are percentages.

*Occupational code is the Danish version of the International Standard Classification of Occupations from 1988 (DISCO-88).

**Table 2:** Pregnancy outcomes and gestational age at recruitment.

	Population
**Pregnancy outcome**	
Live birth	65 161
Fetal death	2717
Induced abortion	
Early	4
Late, maternal indication	14
Late, fetal indication	166
Preterm birth (all)	3128
Induced preterm birth	1475
Loss to follow up*	24
**Timing of recruitment (gestational weeks)**	
<7	5792
7–8	13 842
9–10	17 053
11–12	12 920
13–16	13 631
17–20	3761
21–28	1050
≥29	37

*Loss to follow-up because of emigration and death.

Comparison of self-reported daily loads lifted with JEM-based measures revealed that higher gestational age at interview and higher maternal age were associated with reporting of lower loads lifted per day than estimated based on the JEM (around 10 kg lower). This would suggest that within a given job group, women with higher age or more advanced pregnancy lifted less than the average. Women who reported higher loads lifted per day were more likely to have a pre-pregnancy body mass index ≥30 kg/m^2^, smoke in pregnancy, and be multiparous (e.g. the coefficient for smoking >10 cigarettes per day as compared to no smoking was 32.1 kg; 95% CI 20.3, 43.9). Importantly, data collected retrospectively (i.e., after a fetal death) was associated with reporting of higher loads (coefficient 24.8 kg; 95% CI 6.6, 43.0), which would be consistent with recall bias.

### Fetal death

In total 2,717 fetal deaths were identified in the cohort, Table 2. We found an increased risk of stillbirth in the highest lifting category (201–975 kg/day) – although the result was not significant when adjusted for potential confounders (HR = 1.40; 95% CI 0.92, 2.14), Table 3. There was no exposure-response relationship between occupational lifting and risk of fetal death, no matter if early or late fetal deaths or stillbirths.

**Table 3:** Hazard ratios for fetal death in relation to occupational lifting (N = 68,086).

	Fetal death in the whole period (N = 68,086/2,717 events)					Early fetal death (12 weeks or less, n = 43,782/1,192 events)					Late fetal death (13–21 weeks, n = 66,135/1,184 events)					Stillbirth (22 weeks or more, n = 65,545/341 events)				
	n	Fetal death	HR*	HR**	95% CI	n	Fetal deaths	HR*	HR**	95% CI	n	Fetal deaths	HR*	HR**	95% CI	n	Fetal deaths	HR*	HR**	95% CI
Kilo lifted per day																				
**0–14**	32,348	1261	1	1	-	20,918	559	1	1	-	31,437	555	1	1	-	31,146	147	1	1	-
**15–50**	7,610	251	0.86	0.86	0.75, 0.99	4,863	116	0.89	0.91	0.75, 1.11	7,405	102	0.80	0.80	0.55, 0.98	7,375	33	0.96	0.92	0.63, 1.34
**51–100**	15,848	715	1.20	1.22	1.11, 1.33	10,259	310	1.18	1.23	1.07, 1.42	15,354	316	1.20	1.21	1.05, 1.39	15,182	89	1.28	1.23	0.93, 1.60
**101–200**	8,207	337	1.13	1.13	1.00, 1.28	5,234	145	1.12	1.16	0.96, 1.41	7,969	147	1.10	1.10	0.91, 1.33	7,898	45	1.28	1.14	0.81, 1.61
**201–975**	4,073	153	1.06	1.07	0.90, 1.28	2,508	62	1.02	1.06	0.81, 1.38	3,970	64	0.98	0.98	0.75, 1.28	3,944	27	1.56	1.40	0.92, 2.14
**P-value for trend**					0.117					0.062					0.992					0.285

* Adjusted for maternal age at time of conception.

** Adjusted for maternal age at time of conception, parity, pre-pregnancy body mass index, and in pregnancy: smoking and alcohol consumption.

In the analysis of women with a history of prior fetal death (n = 12,131), we found an almost three-fold increased risk of stillbirth among women who lifted 201–975 kg/day (HR 2.87; 95% CI 1.37, 6.01), Table 4. This particular group of women comprised mostly child-care workers. When the analysis was adjusted for SES, the adjusted HR was 1.90 (95% CI 0.78, 4.64). For previously pregnant women without a prior fetal death, fetal death and heavy lifting were not associated, Table 5. Corroborating these findings, we found a significant interaction *(P = 0.02)* between occupational lifting and prior fetal death. Based on the observed number of fetal deaths in the highest exposure category (201–975 kg) among women with a previous fetal death, we calculated an excess fraction of 11% in this group. Fetal death among primigravid women (n = 25,762) was not associated with occupational lifting (results not shown).

**Table 4:** Hazard ratios for fetal death among women who experienced fetal death before current pregnancy (n = 12,131).

	Fetal death in the whole period (n = 12,131/560 events)					Early fetal death (12 weeks or less, n = 7,594/263 events)					Late fetal death (13–21 weeks, n = 11,719/233 events)					Stillbirth (22 weeks or more, n = 11,595/64 events)				
	n	Fetal deaths	HR*	HR**	95%CI	n	Fetal deaths	HR*	HR**	95%CI	n	Fetal deaths	HR*	HR**	95%CI	n	Fetal deaths	HR*	HR**	95%CI
Kilo lifted per day																				
**0–14**	5793	249	1	1	-	3,622	119	1	1	-	5,593	103	1	1	-	5,548	27	1	1	-
**15–50**	1,270	41	0.76	0.76	0.54, 1.6	768	23	0.88	0.91	0.72, 1.41	1,230	14	0.64	0.63	0.36, 1.10	1,227	4	0.68	0.61	0.21, 1.77
**51–100**	2,815	153	1.30	1.31	1.07, 1.61	1,798	74	1.31	1.38	1.02, 1.85	2,709	63	1.29	1.28	0.93, 1.76	2,671	16	1.26	1.22	0.65, 2.28
**101–200**	1,442	75	1.30	1.25	0.96, 1.63	906	33	1.21	1.23	0.73, 1.83	1,397	36	1.48	1.37	0.92, 2.03	1,370	6	0.98	0.90	0.36, 2.24
**201–975**	811	42	1.33	1.28	0.91, 1.79	500	14	0.98	1.00	0.57, 1.77	790	17	1.24	1.14	0.67, 1.92	779	11	3.12	2.87	1.37, 6.01
**P-value for trend**					0.054					0.129					0.599					0.106

* Adjusted for maternal age at conception.

** Adjusted for maternal age at conception, parity, pre-pregnancy body mass index, and in pregnancy: smoking and alcohol consumption.

**Table 5:** Hazard ratios for fetal death among women who did not experience fetal death before current pregnancy, but have been pregnant previously (n = 30,130).

	Fetal death in the total period (n = 30.130/1.206 events)					Early fetal death (12 weeks or less, n = 18.789/542 events)					Late fetal death (13–21 weeks, n = 29.212/522 events)					Stillbirth (22 weeks or more, n = 28.990/142 events)				
	n	Fetal deaths	HR*	HR**	95%CI	n	Fetal deaths	HR*	HR**	95%CI	n	Fetal deaths	HR*	HR**	95%CI	n	Fetal deaths	HR*	HR**	95%CI
Kilo lifted per day																				
**0–14**	14.182	559	1	1	-	8.937	258	1	1	-	13.763	242	1	1	-	13.644	59	1	1	-
**15–50**	3.243	118	0.94	0.95	0.78, 1.16	2.002	52	0.90	0.92	0.68, 1.24	3.147	49	0.91	0.92	0.68, 1.25	3.136	17	1.26	1.16	0.67, 2.00
**51–100**	6.973	310	1.18	1.22	1.06, 1.40	4.339	140	1.18	1.23	1.01, 1.51	6.740	132	1.16	1.19	0.97, 1.48	6.679	38	1.33	1.24	0.82, 1.87
**101–200**	3.668	154	1.18	1.22	1.02, 1.47	2.272	63	1.08	1.17	0.88, 1.56	3.555	70	1.22	1.27	0.97, 1.68	3.526	21	1.44	1.23	0.73, 2.05
**201–975**	2.064	65	0.90	0.94	0.73, 1.22	1.239	29	0.92	1.00	0.68, 1.48	2.007	29	0.90	0.95	0.64, 1.40	2.005	7	0.87	0.75	0.34, 1.67
**P-value for trend**					0.827					0.469					0.921					0.611

* Adjusted for maternal age at conception.

** Adjusted for maternal age at conception, parity, pre-pregnancy body mass index, and in pregnancy: smoking and alcohol consumption.

### Preterm birth

A total of 3,128 preterm births were registered, of which 88% occurred in gestational weeks 33 to 36. Overall, we found an exposure-response relationship between total load lifted per day and risk of preterm birth *(P-trend = 0.001)* with a HR of around 1.25 for women lifting 101–975 kg per day, Table 6. Findings were quite similar for extremely, very, and moderately preterm birth. Table 7 shows the risk of preterm birth in relation to total load lifted per day among primi- and multigravid women. We found a clear exposure-response relation for primigravid women, reaching a HR of 1.43 for total loads of 201–975 kg/day. The adjusted HR for a one step increase in exposure category was 1.09 (95% CI 1.04, 1.14; *P<0.001*). With further adjustment for SES, the adjusted HR was 1.07 (95% CI 1.02, 1.12; *P = 0.006*). An exposure-response relationship was less evident for multigravid women, Table 7. Previous fetal death did not affect the association between occupational lifting and preterm birth (results not shown). Based on the observed number of preterm births in primigravid women lifting a minimum load of 15 kg per day, the excess fraction of preterm births was 10%. In total, 47% of the preterm births were induced and analyses using spontaneous preterm birth as the outcome (i.e., with censoring in case of induced birth) showed a little stronger association between total load lifted per day and preterm birth, reaching HR of 1.58 (95% CI 1.17, 2.13) among primigravid women lifting total loads of 201–975 kg/day. For induced preterm birth the corresponding HR was 1.28 (95% CI 0.88, 1.85). The excess fraction of spontaneous preterm birth among primigravid women lifting at least 15 kg was 11%.

**Table 6:** Hazard ratios for preterm birth in relation to occupational lifting (n = 65,530).

	All preterm					Extremely preterm (22–27 completed weeks)					Very preterm (28–32 completed weeks)					Moderately preterm (33–36 completed weeks)				
	n	Preterm births	HR*	HR**	95%CI	n	Preterm births	HR*	HR**	95%CI	n	Preterm births	HR*	HR**	95%CI	n	Preterm births	HR*	HR**	95%CI
Kilo lifted per day																				
**0–14**	37137	1383	1.00	1.00	Reference	30958	30	1.00	1.00	Reference	30855	133	1.00	1.00	Reference	30822	1220	1.00	1.00	Reference
**15–50**	7374	348	1.06	1.03	0.92, 1.16	7342	7	0.99	0.95	0.42, 2.16	7315	30	0.95	0.91	0.61, 1.36	7297	311	1.07	1.05	0.93, 1.19
**51–100**	15177	754	1.12	1.12	1.03, 1.23	15102	13	0.90	0.89	0.46, 1.71	15037	74	1.14	1.14	0.86, 1.52	15003	667	1.11	1.13	1.02, 1.24
**101–200**	7898	441	1.26	1.25	1.12, 1.39	7868	14	1.92	1.93	0.99, 3.74	7822	46	1.34	1.31	0.92, 1.85	7785	381	1.19	1.22	1.09, 1.38
**201–975**	3944	202	1.16	1.22	1.05, 1.42	3926	3	0.83	0.88	0.26, 2.95	3897	25	1.47	1.53	0.98, 2.37	3894	174	1.09	1.19	1.01, 1.40
**P-value for trend**					0.001					0.384					0.031					0.001

* Adjusted for maternal age at conception.

** Adjusted for maternal age at conception, nulliparity, conic section, assisted reproduction, pre-pregnancy body mass index, and in pregnancy: smoking and alcohol consumption.

**Table 7:** Hazard ratios for preterm birth in relation to total load lifted per day among primi- and multigravid women.

	Primigravid women (n = 24,898/1455 events)					Multigravid women (n = 40,595/1670 events)				
	n	Preterm births	HR*	HR**	95% CI	n	Preterm births	HR*	HR***	95% CI
Kilo lifted per day										
**0–14**	11,920	640	1.00	1.00	Reference	19,195	740	1.00	1.00	Reference
**15–50**	3,007	174	1.09	1.09	0.92, 1.28	4,363	174	1.03	1.00	0.84, 1.17
**51–100**	5,820	351	1.16	1.16	1.02, 1.33	9,353	403	1.12	1.10	0.98, 1.25
**101–200**	2,993	206	1.34	1.34	1.14, 1.58	4,900	235	1.21	1.17	1.00, 1.36
**201–975**	1,158	84	1.42	1.43	1.13, 1.80	2,784	118	1.08	1.09	0.89, 1.33
**P-value for trend**					0.001					0.134

* Adjusted for maternal age at conception.

** Adjusted for maternal age at conception, conic section, assisted reproduction, pre-pregnancy body mass index, and in pregnancy: smoking and alcohol consumption.

***In addition for nulliparity and previous spontaneous abortion among multigravid women.

¤ Due to missing information on primi-/multigravid status, 37 women were excluded from these analyses.

## Discussion

We studied the risk of fetal death and preterm birth in relation to occupational lifting as assessed by a JEM. For women with a prior fetal death, we found an almost three-fold increased risk of fetal death after week 22 among women lifting 201–975 kg/day. After adjusting for SES the association disappeared, which was expected as SES and exposure are highly correlated. Assuming a causal association, we estimated that 11% of the fetal deaths in this group could be prevented if these women avoided lifting total loads >200 kg/day. For preterm birth, we found an exposure-response relationship for primigravid women, reaching a HR of 1.43 for total loads of 201–975 kg/day, and for this group, we estimated that 10% of preterm births might be prevented by minimizing lifting exposure during pregnancy. Induced preterm births comprised 47% of all preterm births and were less clearly related to occupational lifting. Comparison of individual and JEM-based measures of lifting showed that women reported higher exposures if they were interviewed after a fetal death.

Besides a study population of more than 65,000 occupationally active pregnant women, the present study benefited from linkage of questionnaire data with nationwide registers and nearly complete follow-up. Importantly, the size of the study cohort allowed separate analyses for primigravid women, and we were able to conduct analyses with censoring of induced preterm births. We were also able to perform thorough confounder adjustment. We preferred using a JEM based on prospective data to individual self-reported exposures because we wanted to reduce the impact of individual differences in perception of lifting the same amounts; e.g. women with complications in the existing pregnancy might tend to overestimate their exposures, while being at a higher risk of preterm birth or fetal death. The JEM approach enabled us to allocate exposure measures to all women with known DISCO-88 code, despite incomplete information on lifting activities or retrospective data of lifting activities due to early pregnancy loss resulting in retrospective exposure information. In this way, we minimized the possibility of inflated effect measures due to recall bias. Furthermore, group-based exposure assessment is less subject to underestimation of exposure-response relationships than individual-based exposure assessment because attenuation of risk estimates is less in the former approach [9]. Still, our exposure-response estimates must be expected to be conservative to the extent that we unintentionally mixed job titles with high and low true exposures within the job groups.

This study has several limitations. First, information on lifting was collected only once around gestational week 16. This implied that we underestimated any true effect of lifting to the extent that women reduced or ceased lifting later in pregnancy due to adaptive measures in the workplace and/or early pregnancy leave or sick leave. In fact, our comparisons of individual and JEM-based measures of lifting supported a decreasing trend of lifting exposures with increasing gestational age. Second, we did not have the possibility to validate the JEM-based exposure estimates against estimates obtained by observation or technical measurements. Therefore the indicated exposure thresholds should be interpreted with caution. It seems plausible that relatively older women lift less than younger women within the same job group. It is harder to judge if women who smoke or have a high BMI actually lift higher loads or if they are more likely to overestimate their exposures because they experience them as more physically demanding. Therefore, these variables were not used for further modeling of the JEM-based exposure estimates. Third, the prevalence of fetal death in our sample was only 4%, i.e. lower than prevalence estimates of around 14% recorded by the DNPR with complete national coverage [17]. The low prevalence may be partly explained by late enrollment into the study so that pregnancies ending in early fetal death were not included. Women who experienced a fetal death between the first antenatal care visit and interview may also have been less likely to participate. However, fetal deaths that occurred after interview were identified through the DNPR which has a high validity of records of fetal death [18]. Therefore we believe that we had valid data on fetal death within in the study, but the risk of early fetal death escapes our evaluation.

A recent study based on almost the same cohort as the present study found an increased risk of fetal death during the first 12 weeks of gestation (with a HR of up to 2.02) for women who reported occupational lifting [6]. These results were not corroborated by our JEM-based analyses. We think that most likely, the explanation for this discrepancy is recall bias in the previous study because women who experienced a fetal death or considered their pregnancy to be at an increased risk of fetal death (e.g. due to pregnancy complications) overestimated their lifting exposures. The just-mentioned study [6] estimated the risk of recall bias by comparing the likelihood of reporting any occupational lifting (>10 kg/day) among women interviewed before and after a fetal death, and results were reassuring. However, these analyses disregarded the possibility that the absolute magnitude of recall bias (number of kg) increased with increasing true lifting exposure because women who lifted 10–20 kg/day were less likely to exaggerate their exposure by e.g. 100 kg than women who lifted >500 kg/day. Our comparison of individual and JEM-based measures of lifting corroborated the risk of recall bias in analyses relying on retrospective exposure data.

In cohort studies, it has not been possible to find a clear association between occupational lifting in pregnancy and the risk of fetal death (RR = 0.40–1.14) [12], [19], [20], but the examined total loads lifted per day typically did not exceed 100–200 kg, which means that the studies were not informative with respect to risks from heavy lifting [8]. However, one cohort study found an elevated risk of fetal death among women who had experienced a prior fetal death (n = 5) [19], which supports our result in this subgroup. Results from earlier case-control studies on lifting and fetal death are conflicting; some found an association between heavy lifting and the risk of fetal death (RR = 1.7–3.6) although the single loads lifted varied across studies (7.5–25 kg), just as the lifting frequency (lifting: yes/no, 6–50+ times/day), and others were not able to detect any effect of heavy lifting on fetal death [7]. The just-mentioned recent meta-analysis concluded that women with at risk pregnancies should receive tailored individual counseling [7]; in agreement with this, our results indicated that there is a special need to ensure low lifting exposures for women who have previously experienced a fetal death.

In our previous study, which was based on largely the same dataset, but used individual self-reported exposures [10], we found evidence of an increased risk of extremely preterm birth, with a HR of 4.3 (95% CI 1.4; 13.8) for women lifting more than 1000 kg [10]. In our present study, we did not find this association again, most likely because of our JEM-based approach to minimize recall bias together with the small number of extremely preterm births. The most recent review on preterm birth and occupational lifting concluded that large effects (i.e. RR>1.2) could be effectively ruled out, and a subsequent report from the Netherlands did not find significant associations between preterm birth and self-reported lifting [4], [21]–[23]. However, as was the case for fetal death, we think that the lack of associations may well be explained by low exposure contrasts between those categorized as heavy lifters and those categorized as non-lifters. Moreover, the review and most previous studies did not distinguish between primigravid and multigravid women; our results suggested that it is important to focus on primigravid women because otherwise associations may be underestimated.

In conclusion, our findings based on a JEM with prospectively collected lifting data did not support an exposure-response relationship between occupational lifting of up to 1 ton/day and fetal death. For women with a prior fetal death, the risk of fetal death was almost three-fold increased if they lifted >200 kg/day. Among primigravid women, we found an exposure-response relationship between occupational lifting and preterm birth, reaching a HR of 1.4 for total loads >200 kg/day. About 10% of spontaneous preterm births might be prevented by minimizing lifting exposure in this group, assuming that associations are causal. In spite of reassuring evidence that occupational lifting in general infers a small risk, if any, there seems to be good reason to limit high levels of occupational lifting during pregnancy.

## References

[1] Wilcox AJ (2010) Fertility and Pregnancy - An Epidemiologic Perspective. Oxford New York: Oxford University Press.

[2] Saigal S, Doyle LW (2008) An overview of mortality and sequelae of preterm birth from infancy to adulthood. Lancet 371 (9608) 261–9. 18207020 10.1016/S0140-6736(08)60136-1

[3] Langhoff-Roos J, Kesmodel U, Jacobsson B, Rasmussen S, Vogel I (2006) Spontaneous preterm delivery in primiparous women at low risk in Denmark: population based study. BMJ 332 (7547) 937–9. 16497733 10.1136/bmj.38751.524132.2F PMC1444877

[4] Snijder CA, Brand T, Jaddoe V, Hofman A, Mackenbach JP, et al (2012) Physically demanding work, fetal growth and the risk of adverse birth outcomes. The Generation R Study. Occup Environ Med 69 (8) 543–50. 22744766 10.1136/oemed-2011-100615

[5] Nugteren JJ, Snijder CA, Hofman A, Jaddoe VW, Steegers EA, et al (2012) Work-related maternal risk factors and the risk of pregnancy induced hypertension and preeclampsia during pregnancy. The Generation R Study. PLoS One 7 (6) e39263. 22720087 10.1371/journal.pone.0039263 PMC3376127

[6] Juhl M, Strandberg-Larsen K, Larsen PS, Andersen PK, Svendsen SW, et al (2013) Occupational lifting during pregnancy and risk of fetal death in a large national cohort study. Scand J Work Environ Health 39 (4) 335–42. 23207454 10.5271/sjweh.3335

[7] Bonde JP, Jorgensen KT, Bonzini M, Palmer KT (2013) Miscarriage and occupational activity: a systematic review and meta-analysis regarding shift work, working hours, lifting, standing, and physical workload. Scand J Work Environ Health 39 (4) 325–34. 23235838 10.5271/sjweh.3337 PMC3699369

[8] Palmer KT, Bonzini M, Harris EC, Linaker C, Bonde JP (2013) Work activities and risk of prematurity, low birth weight and pre-eclampsia: an updated review with meta-analysis. Occup Environ Med 70 (4) 213–22. 23343859 10.1136/oemed-2012-101032 PMC3653070

[9] Armstrong BG (1998) Effect of measurement error on epidemiological studies of environmental and occupational exposures. Occup Environ Med 55 (10) 651–6. 9930084 10.1136/oem.55.10.651 PMC1757516

[10] Runge SB, Pedersen JK, Svendsen SW, Juhl M, Bonde JP, et al (2013) Occupational lifting of heavy loads and preterm birth: a study within the Danish National Birth Cohort. Occup Environ Med 70 (11) 782–8. 23839660 10.1136/oemed-2012-101173

[11] Saurel-Cubizolles MJ, Subtil D, Kaminski M (1991) Is preterm delivery still related to physical working conditions in pregnancy? J Epidemiol Community Health 45 (1) 29–34. 2045741 10.1136/jech.45.1.29 PMC1060698

[12] Ahlborg G Jr, Bodin L, Hogstedt C (1990) Heavy lifting during pregnancy–a hazard to the fetus? A prospective study. Int J Epidemiol 19 (1) 90–7. 2351529 10.1093/ije/19.1.90

[13] Lawson CC, Whelan EA, Hibert EN, Grajewski B, Spiegelman D, et al (2009) Occupational factors and risk of preterm birth in nurses. Am J Obstet Gynecol 200 (1) 51–8. 18976732 10.1016/j.ajog.2008.08.006 PMC4249587

[14] Henderson JJ, McWilliam OA, Newnham JP, Pennell CE (2012) Preterm birth aetiology 2004–2008. Maternal factors associated with three phenotypes: spontaneous preterm labour, preterm pre-labour rupture of membranes and medically indicated preterm birth. J Matern Fetal Neonatal Med 25 (6) 642–7. 21827362 10.3109/14767058.2011.597899

[15] Olsen J, Melbye M, Olsen SF, Sorensen TI, Aaby P, et al (2001) The Danish National Birth Cohort–its background, structure and aim. Scand J Public Health 29 (4) 300–7. 11775787 10.1177/14034948010290040201

[16] Braveman PA, Cubbin C, Egerter S, Chideya S, Marchi KS, et al (2005) Socioeconomic status in health research: one size does not fit all. JAMA 294 (22) 2879–88. 16352796 10.1001/jama.294.22.2879

[17] Nybo Andersen AM, Wohlfahrt J, Christens P, Olsen J, Melbye M (2000) Maternal age and fetal loss: population based register linkage study. BMJ 320 (7251) 1708–12. 10864550 10.1136/bmj.320.7251.1708 PMC27416

[18] Lohse SR, Farkas DK, Lohse N, Skouby SO, Nielsen FE, et al (2010) Validation of spontaneous abortion diagnoses in the Danish National Registry of Patients. Clin Epidemiol 2: 247–50. 21152251 10.2147/CLEP.S13815 PMC2998814

[19] Fenster L, Hubbard AE, Windham GC, Waller KO, Swan SH (1997) A prospective study of work-related physical exertion and spontaneous abortion. Epidemiology 8 (1) 66–74. 9116099 10.1097/00001648-199701000-00011

[20] Florack EI, Zielhuis GA, Pellegrino JE, Rolland R (1993) Occupational physical activity and the occurrence of spontaneous abortion. Int J Epidemiol 22 (5) 878–84. 8282467 10.1093/ije/22.5.878

[21] Bonzini M, Coggon D, Palmer KT (2007) Risk of prematurity, low birthweight and pre-eclampsia in relation to working hours and physical activities: a systematic review. Occup Environ Med 64 (4) 228–43. 17095552 10.1136/oem.2006.026872 PMC2078455

[22] Bonzini M, Coggon D, Godfrey K, Inskip H, Crozier S, et al (2009) Occupational physical activities, working hours and outcome of pregnancy: findings from the Southampton Women's Survey. Occup Environ Med 66 (10) 685–90. 19770355 10.1136/oem.2008.043935 PMC3088899

[23] Burdorf A, Brand T, Jaddoe VW, Hofman A, Mackenbach JP, et al (2011) The effects of work-related maternal risk factors on time to pregnancy, preterm birth and birth weight: the Generation R Study. Occup Environ Med 68 (3) 197–204. 21172792 10.1136/oem.2009.046516

